# Supplementary material for: Giant single-crystal-to-single-crystal transformations associated with chiral interconversion induced by elimination of chelating ligands
Source: Nat Commun. 2021 Nov 25;12:6908. doi: 10.1038/s41467-021-27282-9 (PMC8616912; doi:10.1038/s41467-021-27282-9)
Supplement: Supplementary file 1 — Supplementary Information [file 41467_2021_27282_MOESM1_ESM.pdf]

Supplementary Information for:

Giant single-crystal-to-single-crystal transformations associated with chiral interconversion induced by elimination of chelating ligands

Yun Li[1], Bo Zhao[1], Jin-Peng Xue[1],Jing Xie[1], Zi-Shuo Yao[1]*, Jun Tao[1]*

[1]Key Laboratory of Cluster Science of Ministry of Education, School of Chemistry and Chemical Engineering, Beijing Institute of Technology, Beijing 100081, People's Republic of China.

Table of Contents

Supplementary Methods.. S4

Single crystal X-ray diffraction (SXRD).. S4

Powder X-ray diffraction (PXRD) S4

Theoretical calculations... S4

Supplementary Table 1. Crystal data of **1-0D**, **1-1D**, **1-3D**, and **1-0D** (regained)................ S6

Supplementary Table 2. The volumetric shrinkage and weight loss in typical crystalline materials that undergoes reversible solid-state transformations.. S7

Supplementary Fig. 1. The complex cation of **[Zn^{II}(eg)$_3$]$^{2+}$** in Λ and Δ configuration. S8

Supplementary Fig. 2. The strong hydrogen bond interactions between the complex cation and sulfates in the compound of **1-0D**. S9

Supplementary Table 3. Distance (Å) and angles (°) of the O–H···O hydrogen bonds in **1-0D**. S9

Supplementary Fig. 3. The TG curve of compound **1-0D** at different heating rates. S10

Supplementary Fig. 4. The time-lapse photographs of the crystals before, during and after the transformations from **1-0D** to **1-1D**, and further to **1-3D**. S11

Supplementary Fig. 5. The large single crystal of **1-1D** prepared by heating **1-0D** at 366 K for 2 h. S12

Supplementary Fig. 6. The strong hydrogen bond interactions between the complex cation and sulfates in the compound of **1-1D**. S13

Supplementary Table 4. Distance (Å) and angles (°) of the O–H···O hydrogen bonds in **1-1D**. S13

Supplementary Fig. 7. The helixes with opposite handedness in the racemic crystals of **1-1D**. The helixes in each individual single crystal manifest the same handedness. S14

Supplementary Fig. 8. The twisted Λ conformation of complex cations in the **1-0D** are converted into identical *cis*-Δ configuration in the **1-1D** with left handedness. S15

Supplementary Fig. 9. The strong hydrogen bond interactions between the complex cation and

sulfates in the compound of **1-3D**. S16

Supplementary Table 5. Distance (Å) and angles (°) of the O–H···O hydrogen bonds in **1-3D**. S16

Supplementary Fig. 10. The reversibility of **1-3D** to **1-1D** and further to **1-0D** monitored by the in-situ PXRD. S17

Supplementary Fig. 11. The CD spectra of individual single crystals and powder crystals. S18

Supplementary Fig. 12. The face index of single crystal of **1-0D** (left) and **1-1D** (right) performed on the same single crystal. S19

Supplementary Fig. 13. The competition between the left- and right- handedness during the structural contraction leads to the single crystals of **1-1D** with racemic structures. S20

Supplementary Fig. 14. Proposed mechanism for **1-1D** (a) to **1-3D** (b). S21

Supplementary Table 6. Crystal data of **$[Co^{II}(eg)_3]SO_4$** and **$[Co^{II}(eg)_2(\mu\text{-}SO_4)]_n$**. S22

Supplementary Fig. 15. The TG curve of compound **$[Co^{II}(eg)_3]SO_4$**. S23

Supplementary Fig. 16. The structural transformation of 0D **$[Co^{II}(eg)_3]SO_4$** to 1D **$\{[Co^{II}(eg)]_2(\mu\text{-}SO_4)_2\}_n$**. S24

Supplementary Table 7. Crystal data of **$[Cu^{II}(eg)_3]SO_4$** and **$\{[Cu^{II}(eg)]_2(\mu\text{-}SO_4)_2\}_n$**. S25

Supplementary Fig. 17. The TG curve of compound **$[Cu^{II}(eg)_3]SO_4$**. S26

Supplementary Fig. 18. The building block in 0D **$[Cu^{II}(eg)_3]SO_4$** (a) and 1D **$\{[Cu^{II}(eg)]_2(\mu\text{-}SO_4)_2\}_n$** (b). S27

Supplementary Fig. 19. The structural transformation from 0D **$[Cu^{II}(eg)_3]SO_4$** to 1D **$\{[Cu^{II}(eg)]_2(\mu\text{-}SO_4)_2\}_n$**. S28

Supplementary Table 8. Comparison between experimental (exp.) and calculated (calc.) lattice parameters of the structures from **1-0D** to **1-1D**. S29

Supplementary Table 9. Calculated Gibbs free energies of each structure in unit cell with zero-point energy correction and entropy correction at 378 K. S29

Supplementary Methods

Single crystal X-ray diffraction (SXRD): SXRD analyses of samples were performed on a Rigaku XtaLab Pro diffractometer equipped with a Mo Kα radiation source. The temperature of sample was controlled with a continuous cold N_2 gas flow. The data of **1-0D** and **1-1D** were collected with one sample in the following order: the data of **1-0D** was collected at 278 K, then the crystal was in-situ heated to 366 K and maintained for 2 h, after which the SC-XRD of **1-1D** were performed at 372 K. The crystal data of **1-3D** were collected at 273 K. The structures were solved by a direct method and refined via full-matrix least-squares on F^2 using the SHELX program[1] with anisotropic thermal parameters for all non-hydrogen atoms. The hydrogen atoms were geometrically added and refined by the riding model. The structures of $[Co^{II}(eg)_2(\mu\text{-}SO_4)]_n$ and $\{[Cu^{II}(eg)]_2(\mu\text{-}SO_4)_2\}_n$ were refined with TWIN and BASF instructions because the twin domains formed during the symmetry-breaking structural transformations.

Powder X-ray diffraction (PXRD): PXRD was recorded on a PANalytical diffractometer with Cu Kα radiation equipped with a TTK450 accessory in a temperature range from 273 K to 473 K at an interval of 25 K in a vacuum condition. The sample was stabilized at each temperature point for 30 min before measurement. The time-dependent reverse structural transformation was monitored by immersing the sample of **1-3D** in a mixed solvent of eg and acetone (v/v = 1/8) for different time.

Theoretical calculations: In order to investigate formation process from **1-0D** to **1-1D**, we performed first-principle calculation using the Vienna Ab-initio Simulation Package (VASP).[2] The generalized gradient approximation (GGA)[3] with the Perdew-Burke-Ernzerhof (PBE) is

employed to describe all exchange and correlation effects. The vdW interaction is accounted using DFT-D3 corrections. [4,5] The projector augmented wave (PAW) method [6,7] is used to describe the electrons-ionic interactions. A cutoff energy of 820 eV was chosen for the plane-wave basis to ensure the precision of calculations. Brillouin zone is sampled by a 3 × 3 × 1 k-point grid. The relaxation of the electronic degrees of freedom is assumed to be converged when the total energy changes between the two electronic optimization steps is smaller than 1×10^{-5} eV, and the forces below 0.02 eV/Å for ions are used as the criterion for relaxation convergence. The calculated structures are in excellent agreement with experiment (Supplementary Table 8). The structures are optimized followed by frequency calculations. Gibbs free energies (Supplementary Table 9) are computed by $G = E + ZPE - TS$, where E is electronic energy, ZPE is zero-point energy, S is entropy, T is temperature and we use 378 K to match experimental condition. Since each primitive cell contains four Zn-motif, the energies reported are divided by four to represent the energetics change per Zn-motif.

Supplementary Table 1. Crystal data of **1-0D, 1-1D, 1-3D,** and **1-0D** (regained).

Compound	**1-0D**	**1-1D** (right-hand)	**1-1D** (left-hand)	**1-3D**	**1-0D** (regained)
Empirical formula	$C_6H_{18}O_{10}SZn$	$C_4H_{12}O_8SZn$	$C_4H_{12}O_8SZn$	$C_2H_6O_6SZn$	$C_6H_{18}O_{10}SZn$
Formula weight	347.63	285.57	285.57	223.50	347.63
Temperature/K	293(2)	372(3)	372(1)	270(2)	292(2)
Crystal color	colorless	colorless	colorless	colorless	colorless
Crystal system	monoclinic	orthorhombic	orthorhombic	monoclinic	monoclinic
Space group	$P2_1/c$	$P2_12_12_1$	$P2_12_12_1$	$P2_1$	$P2_1/c$
a/Å	8.7864(4)	6.4536(5)	6.4759(7)	10.0682(5)	8.7665(5)
b/Å	7.6102(3)	9.8866(6)	9.8926(10)	9.9679(4)	7.5918(4)
c/Å	19.3284(10)	15.1208(9)	15.1211(19)	12.7621(6)	19.2946(12)
α/°	90	90	90	90	90
β/°	99.475(5)	90	90	90.093(4)	99.387(6)
γ/°	90	90	90	90	90
Volume/Å^3	1274.79(10)	964.77(11)	968.7(2)	1280.79(11)	1266.93(13)
Z	4	4	4	8	4
Dcalc. / mg·m^{-3}	1.811	1.966	1.958	2.318	1.823
2θ range /°	7.214 to 59.422	8.008 to 59.65	6.844 to 57.194	7.556 to 59.462	6.862 to 56.384
Reflections collected	15216	8028	5978	12623	6495
Independent reflections	3251	2437	2102	5776	2804
R(int)	0.0739	0.0428	0.0498	0.0356	0.0684
Completeness	99.2%	99.4%	99.7%	98.8%	99.8%
Data/restraints/parameters	3251/18/182	2437/12/139	2102/12/136	5776/25/385	2804/18/181
Goodness-of-fit on F^2	0.999	1.021	1.069	0.997	1.012
Final R indices [I>2sigma(I)]	$R_1 = 0.0407$ $\omega R_2 = 0.1037$	$R_1 = 0.0279$ $\omega R_2 = 0.0632$	$R_1 = 0.0421$ $\omega R_2 = 0.0951$	$R_1 = 0.0285$ $\omega R_2 = 0.0623$	$R_1 = 0.0576$ $\omega R_2 = 0.1504$
R indexes (all data)	$R_1 = 0.0492$ $\omega R_2 = 0.1092$	$R_1 = 0.0318$ $\omega R_2 = 0.0643$	$R_1 = 0.0531$, $\omega R_2 = 0.0981$	$R_1 = 0.0325$, $\omega R_2 = 0.0640$	$R_1 = 0.0677$ $\omega R_2 = 0.1576$
Flack	--------	0.014(10)	0.01(2)	-0.010(7)	--------
Largest diff. peak and hole / e Å^{-3}	0.67/-0.94	0.38/-0.41	0.51/-0.76	0.54/-0.60	1.43/-1.56

The SC-XRD analyses of **1-0D** and **1-1D** using the same crystal were successively performed

at 293 and 372 K. The high-quality single crystal of **1-0D** regained from **1-3D** confirms the reversibility of giant SCSC transformation.

Supplementary Table 2. The volumetric shrinkage and weight loss in typical crystalline materials that undergoes reversible solid-state transformations.

Compounds	Volumetric shrinkage (%)	Weight loss (%)	Ref.
**Guest sorption**			
ZnGGH-1·(DMF-H_2O)	16	33	8
Py_6Mes	14	~	9
$Li_9K_7W_1Co_{10}[H_2P_8W_{48}O_{186}]\cdot 132H_2O$	31	15	10
$[Cu(SiF_6)(L)_2]\cdot xMeOH\cdot yH_2O$	36	14	11
HYF (tripeptide)*	25	60	12
Ferritin *	51	~	13
**Reaction between guests and hosts**			
$[Co_2(bipy)_3(SO_4)_2(H_2O)_2]$ (bipy)(CH_3OH)	3.4	13	14
$[\{Fe^{III}(Tp)(CN)_3\}_4\{Fe^{II}(MeCN)(H_2O)_2\}_2]\cdot 10H_2O\cdot 2MeCN$	11	21	15
$Cu_2(OH)(C_8H_3O_7S)(H_2O)\cdot 2H_2O$	20	12	16
$CoCl_2$(1,4-dioxane) $(H_2O)_2$	22	14	17
$[Co_{1.5}(tipb)(SO_4)(pta)_{0.5}]\cdot(DMF)_{1.75}$	3.9	7.5	18
$[Mn(L)(H_2O)]_2[Mo(CN)_7]\cdot 2H_2O$	5.8	5.8	19
**$[Zn^{II}(eg)_3]SO_4$**	**50**	**36**	**This work**
**$[Cu^{II}(eg)_3]SO_4$**	**50**	**36**	**This work**

* The hyper-expansion of the lattice damages the single-crystal feature of these crystals composed of biomolecules.

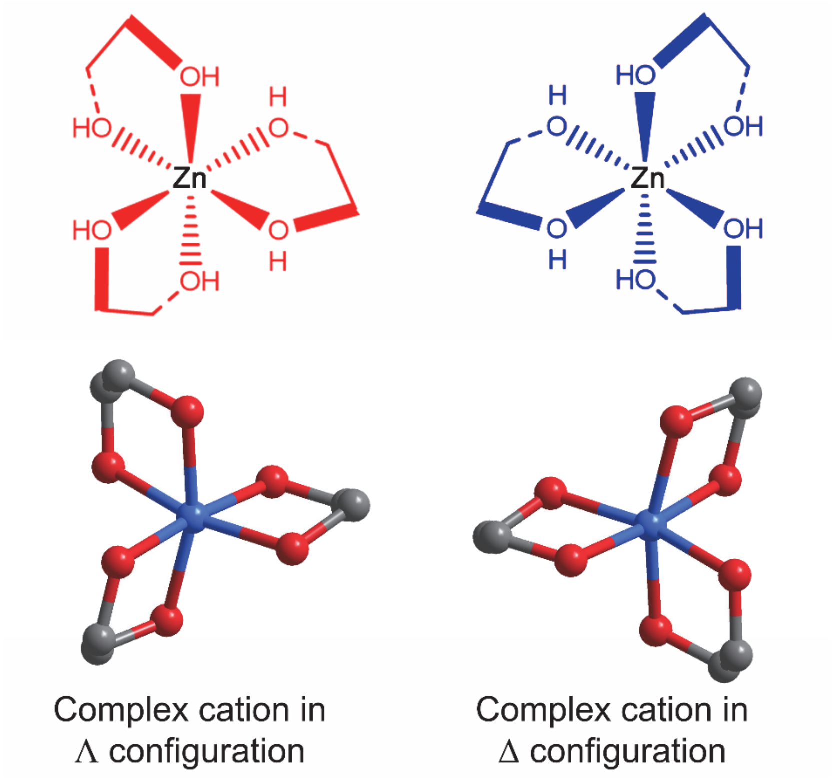

Supplementary Fig. 1. The complex cation of $[Zn^{II}(eg)_3]^{2+}$ in Λ and Δ configuration.

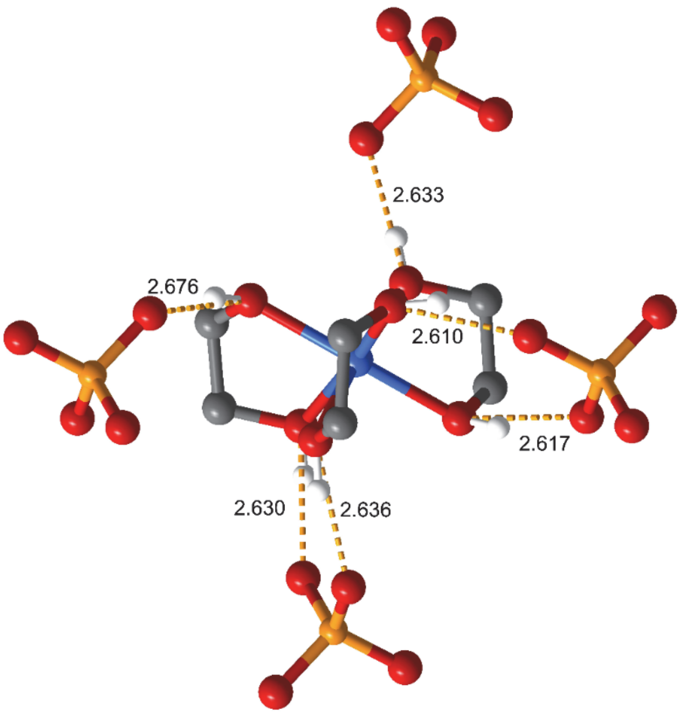

Supplementary Fig. 2. The strong hydrogen bond interactions between the complex cation and sulfates in the compound of 1-0D.

Supplementary Table 3. Distance (Å) and angles (°) of the O–H···O hydrogen bonds in **1-0D**.

D–H	d(D···A)	∠DHA	A
O1–H1	2.610	157.26	O9 [2-x, 1-y, 1-z]
O2–H2	2.636	165.73	O8 [x, y, z]
O3–H3	2.629	159.27	O7 [x, y, z]
O4–H4	2.676	167.10	O10 [1-x, 1-y, 1-z]
O5–H5	2.633	168.50	O10 [x, 1.5-y, -0.5+z]
O6–H6	2.617	162.02	O8 [2-x, 1-y, 1-z]

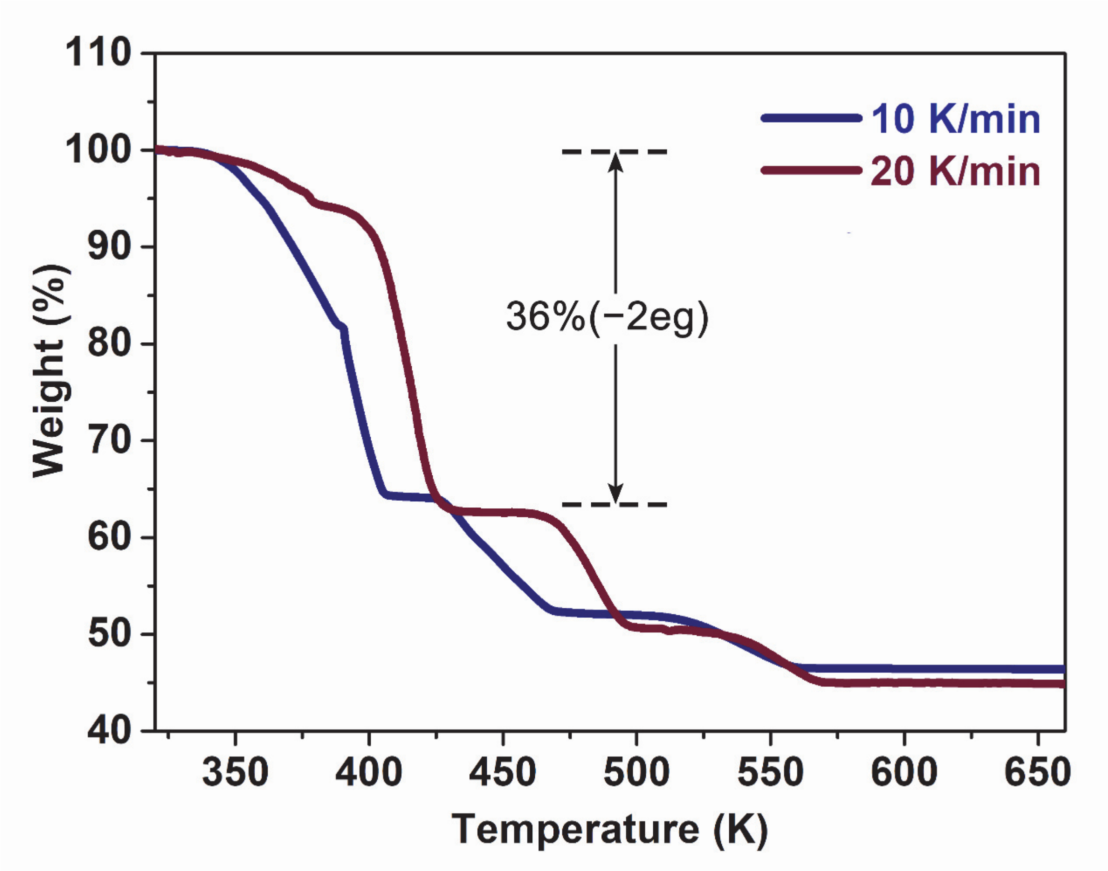

Supplementary Fig. 3. The TG curve of compound 1-0D at different heating rates. The first-step weight loss is largely suppressed when the heating rate is faster than 10 K/min.

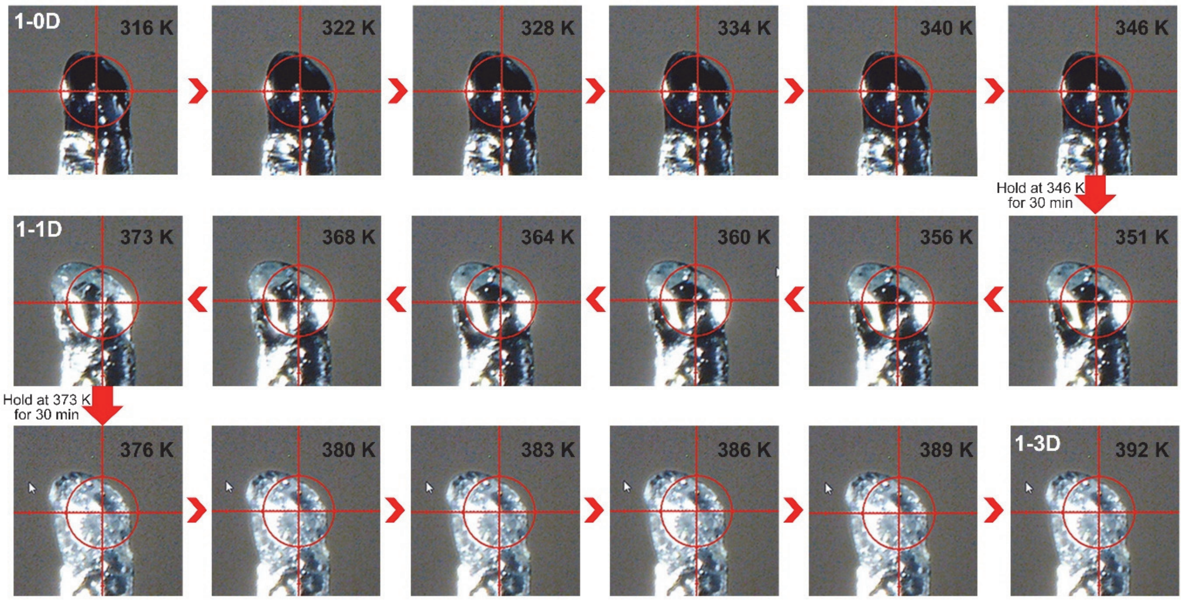

Supplementary Fig. 4. The time-lapse photographs of the crystals before, during and after the transformations from 1-0D to 1-1D, and further to 1-3D. The crystalline shape of the sample retained during the phase transition, eliminating the possibility of crystal dissolution and regrowth during structural transformation.

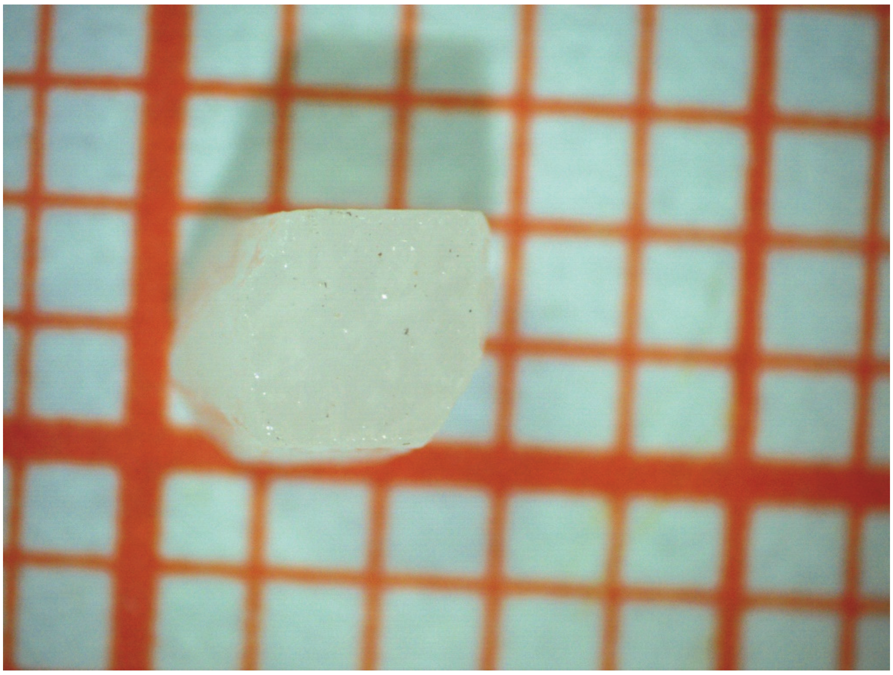

Supplementary Fig. 5. The large single crystal of 1-1D prepared by heating 1-0D at 366 K for 2 h. The individual single crystal was used for CD measurements.

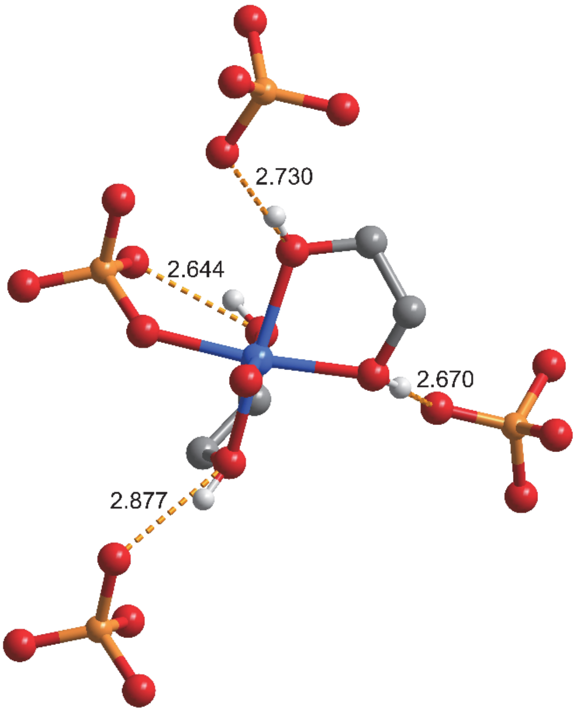

Supplementary Fig. 6. The strong hydrogen bond interactions between the complex cation and sulfates in the compound of 1-1D.

Supplementary Table 4. Distance (Å) and angles (°) of the O–H···O hydrogen bonds in **1-1D**.

D–H	d(D···A)	∠DHA	A
O1–H1	2.877	160.57	O5 [-0.5+x, 1.5-y, 1-z]
O2–H2	2.644	131.01	O7 [1-x, -0.5+y, 1.5-z]
O3–H3	2.669	165.20	O8 [1+x, y, z]
O4–H4	2.730	170.73	O6 [0.5+x, 1.5-y, 1.5-z]

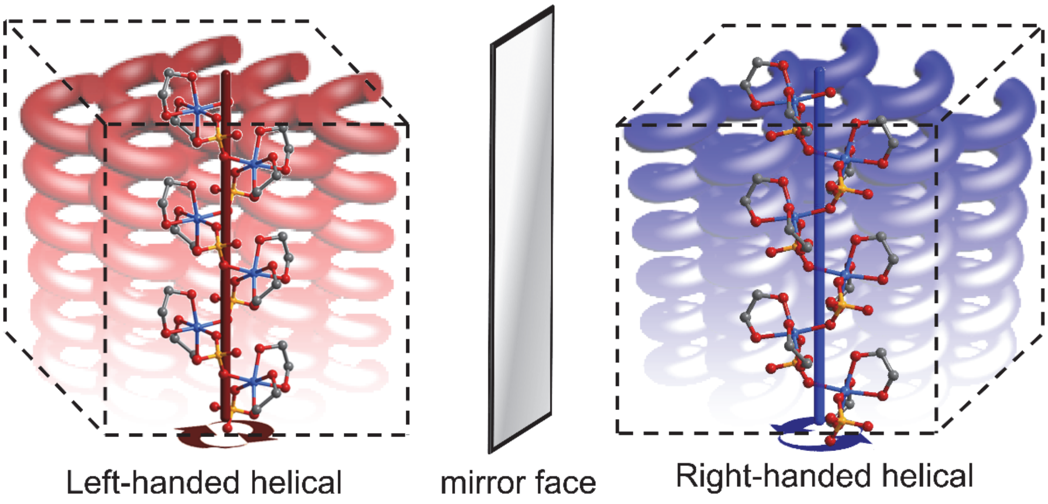

Supplementary Fig. 7. The helixes with opposite handedness in the racemic crystals of 1-1D. The helixes in each individual single crystal manifest the same handedness.

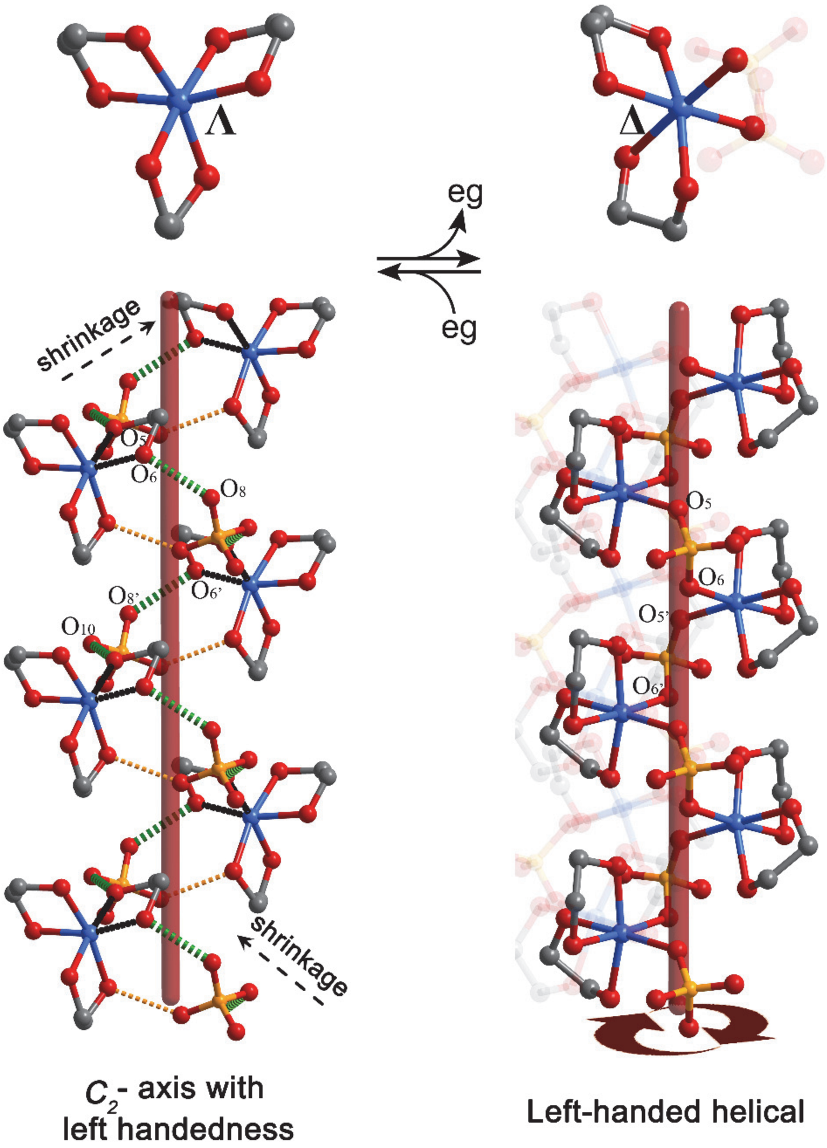

Supplementary Fig. 8. The twisted Λ conformation of complex cations in the 1-0D are converted into identical *cis*-Δ configuration in the 1-1D with left handedness.

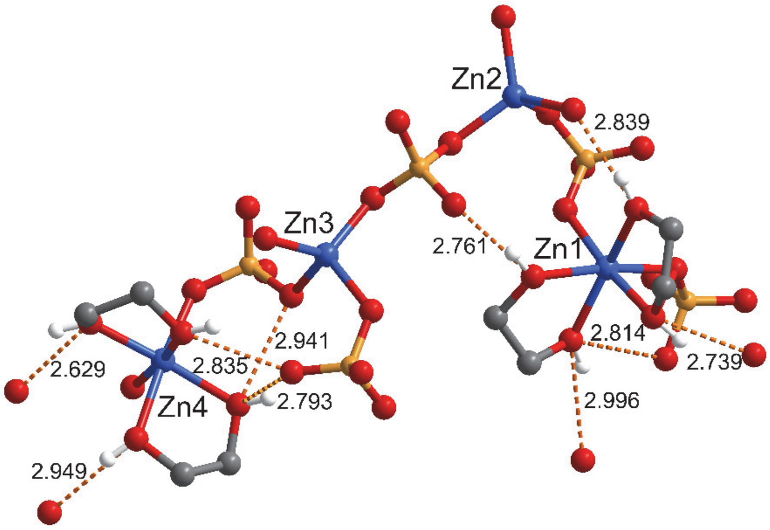

Supplementary Fig. 9. The strong hydrogen bond interactions between the complex cation and sulfates in the compound of 1-3D.

Supplementary Table 5. Distance (Å) and angles (°) of the O–H···O hydrogen bonds in **1-3D**.

D–H	d(D···A)	∠DHA	A
O1–H1	2.839	154.61	O11[2-x, -0.5+y, -z]
O2–H2	2.739	150.28	O24[x, y, z]
O3–H3	2.996	140.18	O14[1-x, 0.5+y, 1-z]
O3–H3	2.814	121.03	O20[2-x, 0.5+y, 1-z]
O4–H4	2.761	165.81	O14[x, y, 1+z]
O5–H5	2.793	130.96	O19[-1+x, y, 1+z]
O5–H5	2.941	108.52	O21[x, y, 1+z]
O6–H6	2.949	172.06	O18[1-x, 0.5+y, 2-z]
O7–H7	2.835	125.87	O19[-1+x, y, 1+z]
O8–H8	2.629	141.50	O10[-1+x, y, 2+z]

The strong hydrogen bond interactions in both the compound **1-0D**, **1-1D** and **1-3D** fasten the crystal lattice and then guarantee the single crystal nature during the giant SCSC transformation.

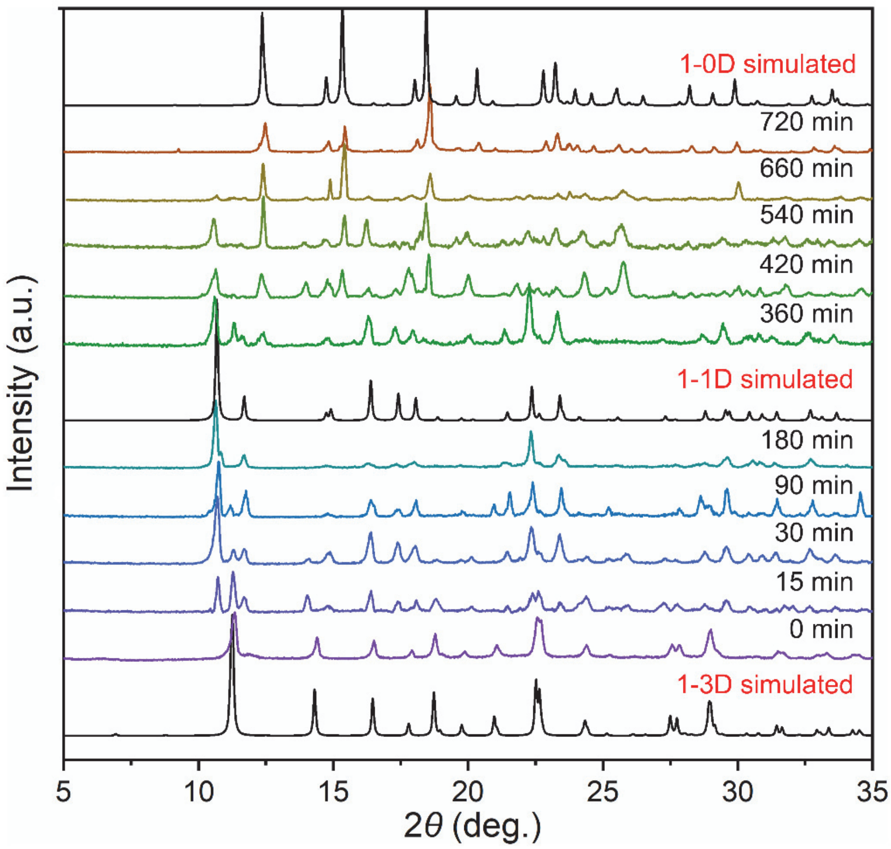

Supplementary Fig. 10. The reversibility of 1-3D to 1-1D and further to 1-0D monitored by the in-situ PXRD. The peaks that denote to the structure of **1-1D** and **1-0D** appear after 180 and 720 min in the acetone solution of eg, respectively, verifying the reversibility of two-step structural transformation. Notably, the time consumption for the structural transition from **1-3D** to **1-1D**, and further to **1-0D** depends on the size and quality of the sample.

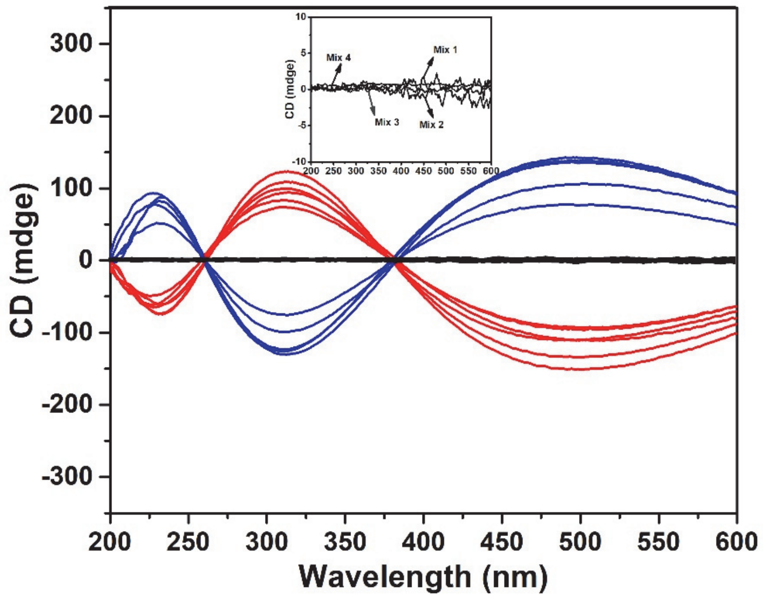

Supplementary Fig. 11. The CD spectra of individual single crystals and powder crystals. The number of individual single crystals with Λ molecular conformation (blue lines) tends to equate to that of Δ molecular conformation (red lines). No distinct CD signal was detected in the powder crystals prepared from a lot of single crystals. These results suggest the bulk state of **1-1D** are racemic mixture that has equal amounts of left- and right-handed enantiomers.

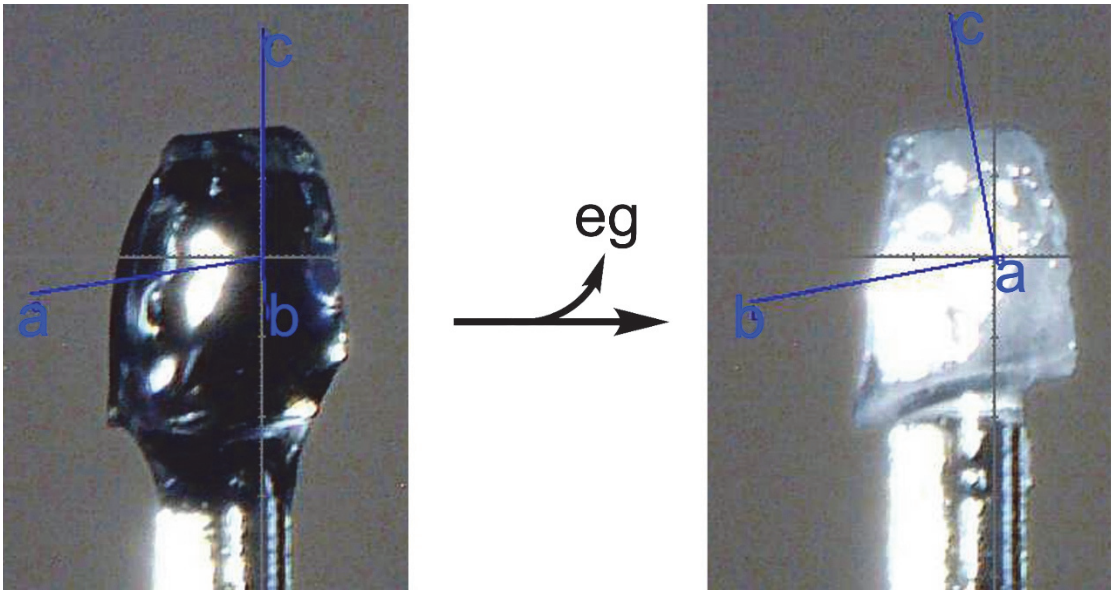

Supplementary Fig. 12. The face index of single crystal of 1-0D (left) and 1-1D (right) performed on the same single crystal. The single crystal of **1-1D** was prepared by heating **1-0D** at 366 K for 2 h. During the first-step SCSC transformation, the crystallographic *b* axis in **1-0D** is converted to crystallographic *a* axis in **1-1D** that is parallel with the 1D coordination helixes.

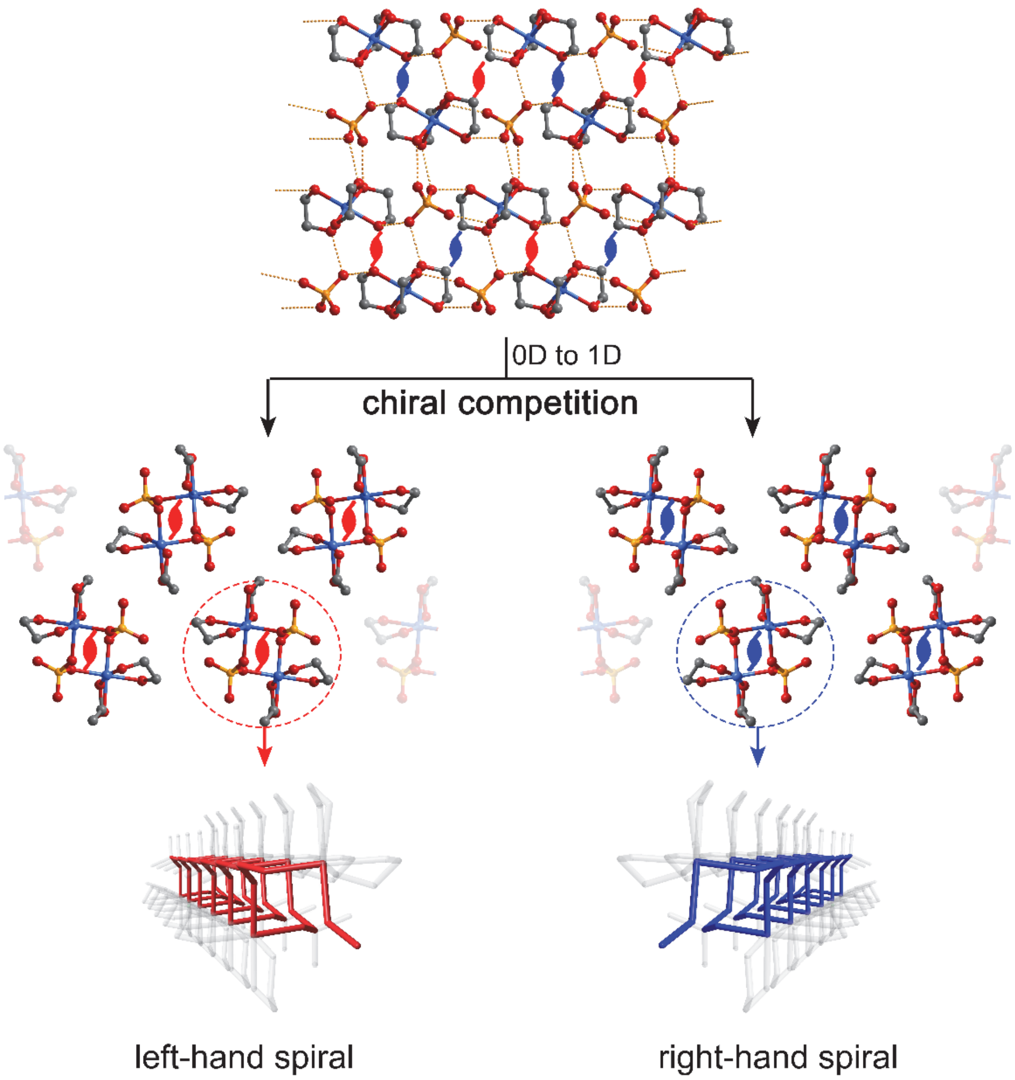

Supplementary Fig. 13. The competition between the left- and right- handedness during the structural contraction leads to the single crystals of 1-1D with racemic structures.

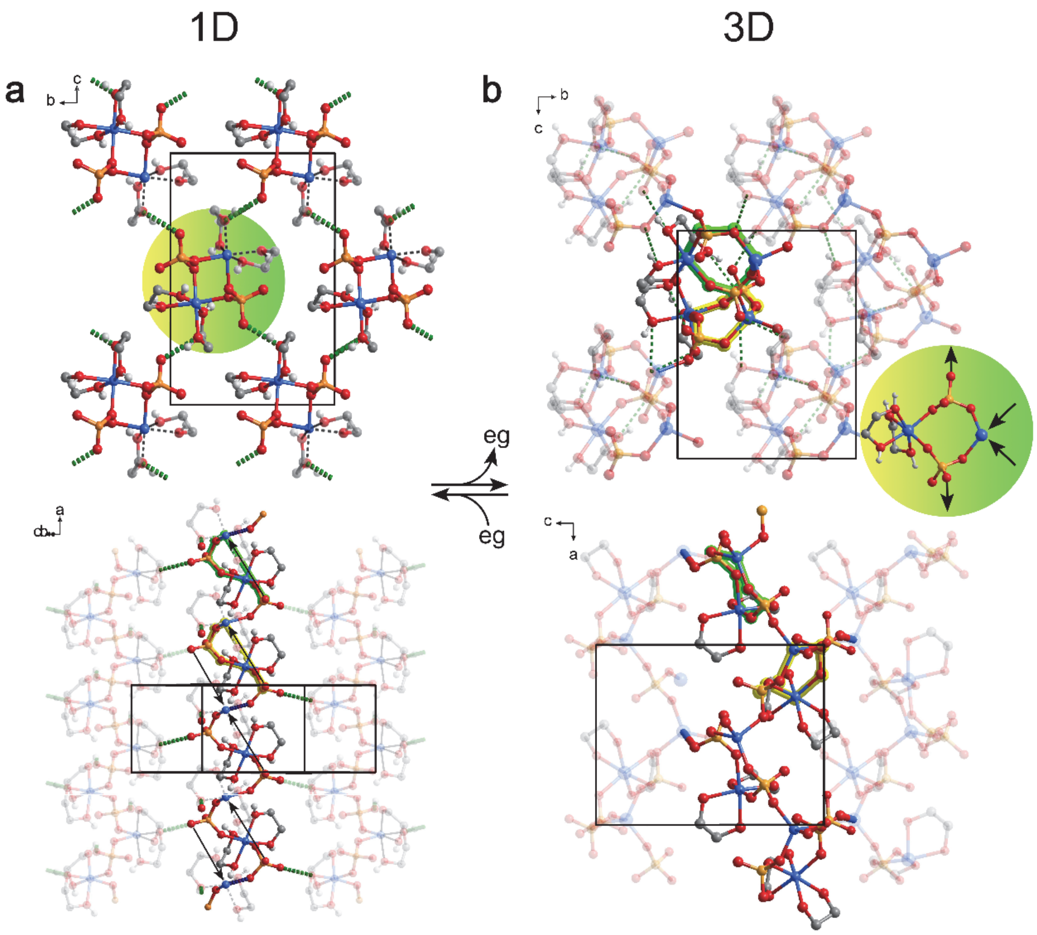

Supplementary Fig. 14. Proposed mechanism for 1-1D (a) to 1-3D (b). The black arrows in down panel indicate the O atoms from sulfate dianions in the adjacent thread shift toward the Zn^{II} metal centers and then form the circular building blocks (inset in the top panel b). The circular building block is composed of one hexacoordinated Zn^{II} ion and one tetracoordinated Zn^{II} bridged by two sulfate anions. It provides two O coordination atoms and two Zn^{II} coordination sites to further connect the other four building blocks around. The dashed green lines denote the intermolecular hydrogen-bond interactions. In the top panel of a, the eg ligands in transparency are eliminated upon heating the crystal to 410 K.

Supplementary Table 6. Crystal data of **$[Co^{II}(eg)_3]SO_4$** and **$[Co^{II}(eg)_2(\mu\text{-}SO_4)]_n$**.

Compound	$[Co^{II}(eg)_3]SO_4$	$[Co^{II}(eg)_2(\mu\text{-}SO_4)]_n$
Empirical formula	$C_6H_{18}O_{10}SCo$	$C_4H_{12}O_8SCo$
Formula weight	341.19	279.13
Temperature/K	298(2)	298(2)
Crystal color	dark-purple	purple
Crystal system	monoclinic	orthorhombic
Space group	$P2_1/c$	$P2_12_12_1$
a/Å	8.8234(5)	6.4943(4)
b/Å	7.5950(4)	9.9222(5)
c/Å	19.2916(9)	15.1193(8)
α/°	90	90
β/°	99.448(5)	90
γ/°	90	90
Volume/$Å^3$	1275.27(12)	974.25(9)
Z	4	4
Dcalc./$mg \cdot m^{-3}$	1.777	1.903
μ/mm^{-1}	1.552	1.994
F_{000}	708.0	572.0
2θ range /°	7.12 to 52.742	7.5 to 59.608
Reflections collected	9904	7334
Independent reflections	2593	2462
R(int)	0.0263	0.0371
Completeness	99.5%	99.5%
Data/restraints/parameters	2593/18/181	2462/12/140
Goodness-of-fit on F^2	1.082	1.056
Final R indices [I>2sigma(I)]	$R_1 = 0.0294$, $\omega R_2 = 0.0754$	$R_1 = 0.0307$, $\omega R_2 = 0.0800$
R indexes (all data)	$R_1 = 0.0326$, $\omega R_2 = 0.0770$	$R_1 = 0.0323$, $\omega R_2 = 0.0807$
Flack	--------	0.48(2)
Largest diff. peak and hole / e $Å^{-3}$	1.22/-0.33	0.42/-0.52

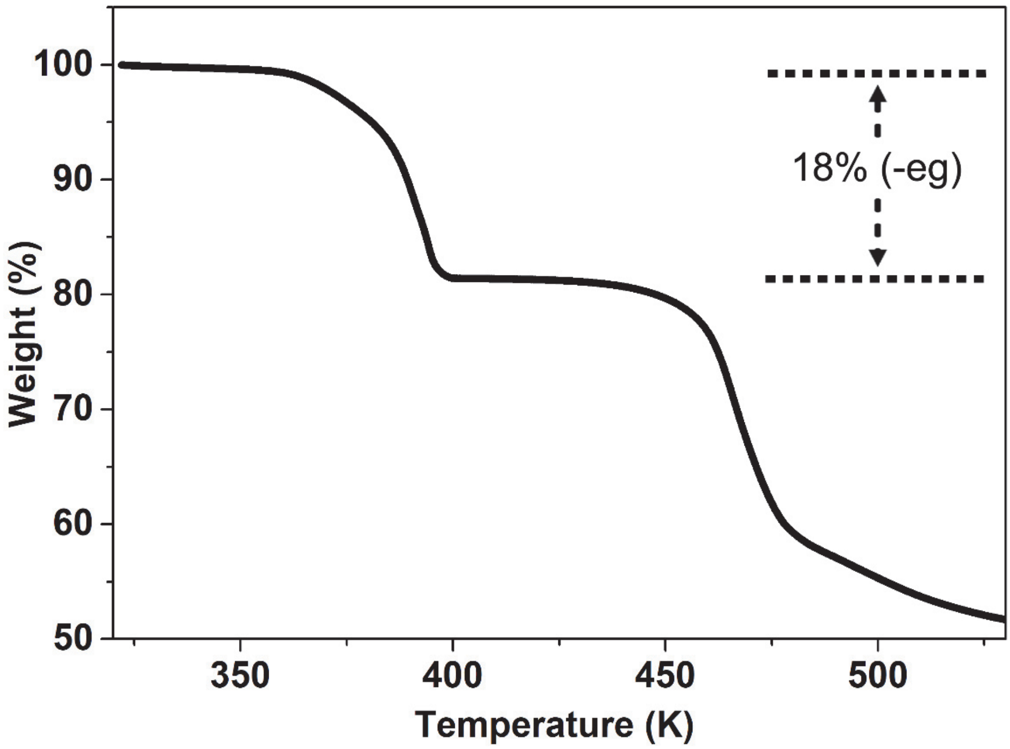

Supplementary Fig. 15. The TG curve of compound $[Co^{II}(eg)_3]SO_4$. Only the first-step structural transformation (0D to 1D) that corresponds to elimination of one third of eg molecules was found in the analog of Ni^{II} and Co^{II}.

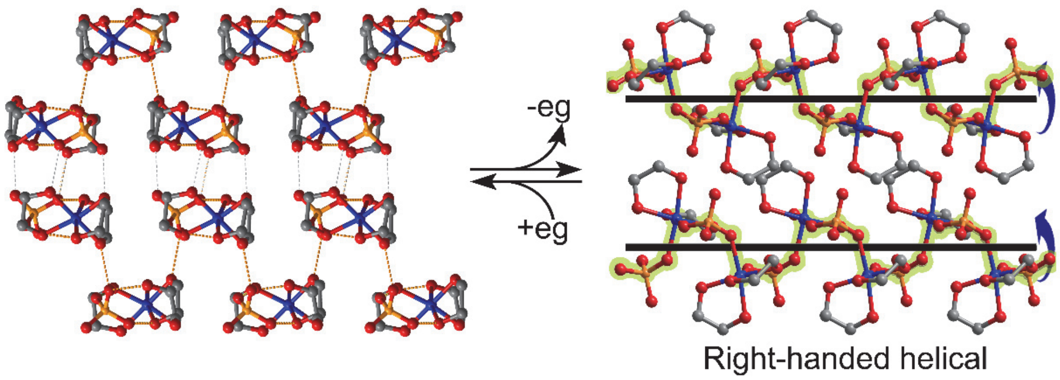

Supplementary Fig. 16. The structural transformation of 0D [Co^{II}(eg)$_3$]SO$_4$ to 1D {[Co^{II}(eg)]$_2$(μ-SO$_4$)$_2$}$_n$.

Supplementary Table 7. Crystal data of **[Cu^{II}(eg)$_3$]SO$_4$** and **{[Cu^{II}(eg)]$_2$(μ-SO$_4$)$_2$}$_n$**.

Compound	[Cu^{II}(eg)$_3$]SO$_4$	{[Cu^{II}(eg)]$_2$(μ-SO$_4$)$_2$}$_n$
Empirical formula	$C_6H_{18}CuO_{10}S$	$C_4H_{12}Cu_2O_{12}S_2$
Formula weight	345.80	443.34
Temperature/K	298 (2)	298 (2)
Crystal color	Light-blue	Light-green
Crystal system	monoclinic	monoclinic
Space group	$P2_1/n$	$P2_1$
a/Å	10.1321(7)	5.1583(4)
b/Å	8.9661(5)	16.3247(14)
c/Å	14.3022(8)	7.5800(6)
α/°	90	90
β/°	104.400(6)	97.013(7)
γ/°	90	90
Volume/Å^3	1258.48(13)	633.52(9)
Z	4	2
Dcalc. / mg·m^{-3}	1.825	2.324
μ/mm^{-1}	1.944	3.747
F_{000}	716.0	444.0
2θ range /°	7.434 to 59.088	7.366 to 50.046
Reflections collected	11601	4948
Independent reflections	3168	2130
R(int)	0.0268	0.0520
Completeness	99.7%	98.5%
Data/restraints/parameters	3168/18/181	2130/60/188
Goodness-of-fit on F^2	1.096	1.135
Final R indices [I>2sigma(I)	R_1 = 0.0258, ωR_2 = 0.0663	R_1 = 0.0752, ωR_2 = 0.2016
R indexes (all data)	R_1 = 0.0290, ωR_2 = 0.0675	R_1 = 0.0760, ωR_2 = 0.2022
Flack	--------	0.43(6)
Largest diff. peak and hole / e Å^{-3}	0.58/-0.44	3.66/-1.09

Notably, the crystal twinning was detected in the structures [Co^{II}(eg)$_2$(μ-SO$_4$)]$_n$ and {[Cu^{II}(eg)]$_2$(μ-SO$_4$)$_2$}$_n$ because of the competition between the left- and right-handedness of enantiomeric structures during symmetry-breaking structural transformation[20,21].

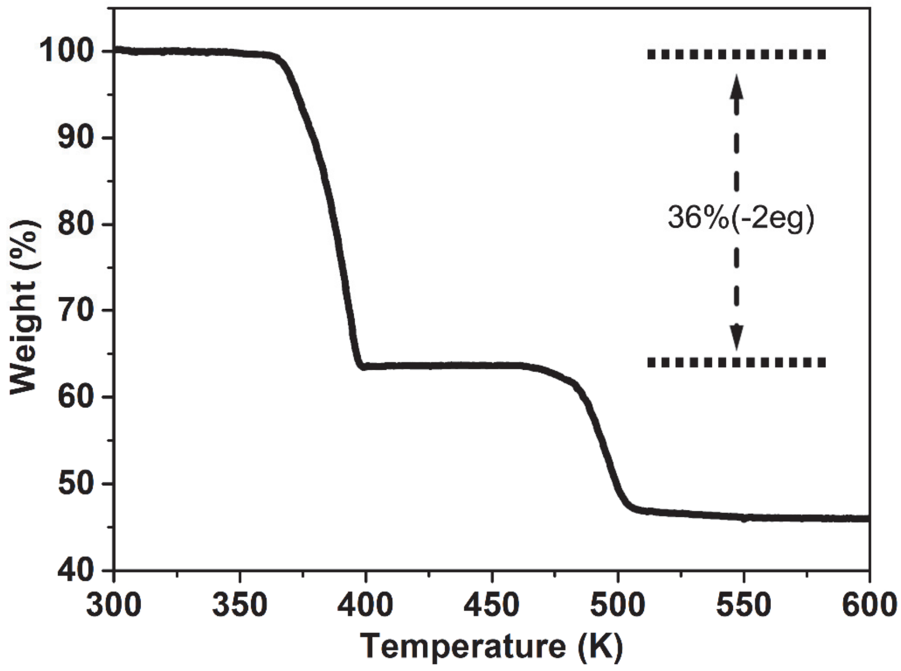

Supplementary Fig. 17. The TG curve of compound [Cu^{II}(eg)$_3$]SO$_4$. Two thirds of eg ligands are collectively removed by heating the crystal to 395 K.

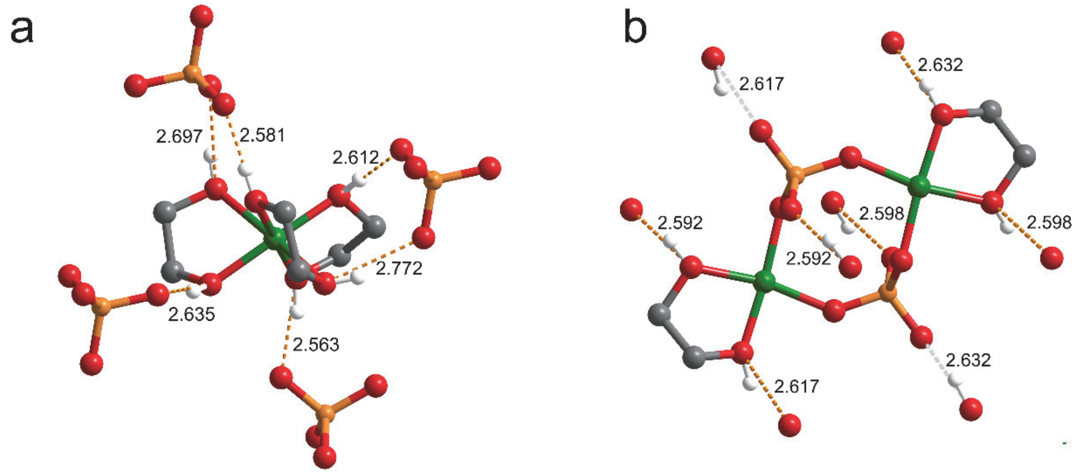

Supplementary Fig. 18. The building block in 0D [Cu^{II}(eg)$_3$]SO$_4$ (a) and 1D {[Cu^{II}(eg)]$_2$(*μ*-SO$_4$)$_2$}$_n$ (b). The sulfate dianions and complex cations are connected through strong hydrogen bonds.

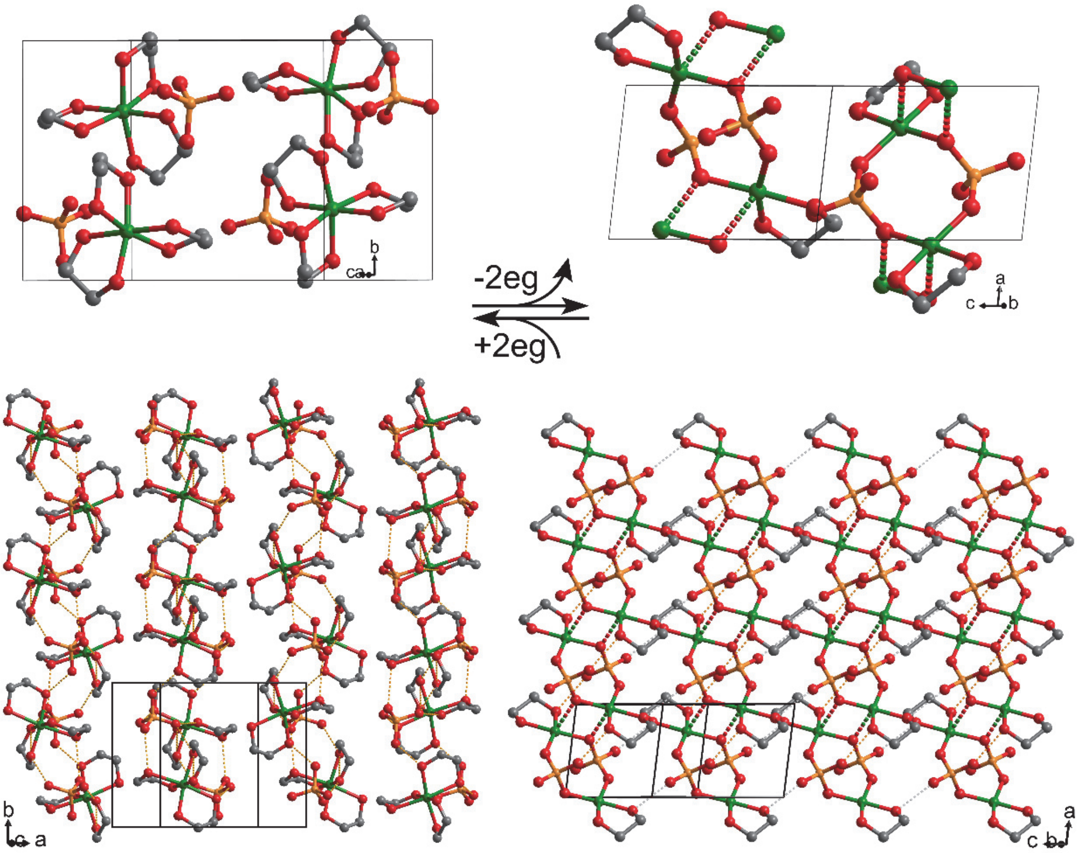

Supplementary Fig. 19. The structural transformation from 0D [Cu^{II}(eg)$_3$]SO_4 to 1D {[Cu^{II}(eg)]$_2$(μ-SO_4)$_2$}$_n$.

Supplementary Table 8. Comparison between experimental (exp.) and calculated (calc.) lattice parameters of the structures from **1-0D** to **1-1D.**

	α (°)	β (°)	γ (°)	a(Å)	b(Å)	c(Å)
1-0D (calc.)	90.0	99.1	90.0	8.78	7.61	19.23
1-0D (exp.)	90.0	99.5	90.0	8.79	7.61	19.32
INT1 (calc.)	91.4	94.8	88.8	7.97	8.54	19.39
INT2 (calc.)	90.2	104.7	89.9	8.04	8.45	19.38
INT3 (calc.)	90.0	90.1	90.0	6.45	9.75	15.81
1-1D (calc.)	90.0	90.0	90.0	6.41	9.95	15.08
1-1D (exp.)	90.0	90.0	90.0	6.45	9.89	15.12

Supplementary Table 9. Calculated Gibbs free energies of each structure in unit cell with zero-point energy correction and entropy correction at 378 K.

structure	chemical formula of unit cell	Gibbs free energy (eV)
1-0D	$4\cdot[Zn\ (C_2H_6O_2)_3]SO_4$	-763.05
INT1	$4\cdot[Zn\ (C_2H_6O_2)_3]\ SO_4$	-758.97
INT2	$4\cdot[Zn\ (C_2H_6O_2)_2]\ SO_4$	-550.32
INT3	$4\cdot[Zn\ (C_2H_6O_2)_2]\ SO_4$	-550.92
1-1D	$4\cdot[Zn\ (C_2H_6O_2)_2]\ SO_4$	-553.52
ethylene glycol	$C_2H_6O_2$	-52.34

Supplementary References

1 Sheldrick, G. M. *et al.* Crystal structure refinement with SHELXL. *Acta Cryst. C.* **71**, 3-8 (2015).

2 Hafner, J. *et al.* Ab-initio simulations of materials using VASP: density-functional theory and beyond. *J. Comput. Chem.* **29** (13), 2044–2078 (2008).

3 Perdew, J. P. *et al.* Generalized gradient approximation made simple. *Phys. Rev. Lett.* **77** (18), 3865-3868 (1996).

4 Grimme, S. *et al.* A consistent and accurate ab initio parametrization of density functional dispersion correction (DFT-D) for the 94 elements H-Pu. *J. Chem. Phys.* **132**, 154104 (2010).

5 Grimme, S. *et al.* Effect of the damping function in dispersion corrected density functional theory. *J. Comput. Chem.* **32**, 1456−1465 (2011).

6 Kresse, G. *et al.* From ultrasoft pseudopotentials to the projector augmented-wave method. *Phys. Rev. B: Condens. Matter. Mater. Phys.* **59**, 1758−1775 (1999).

7 Blochl, P. E. *et al.* Projector augmented-wave method. *Phys. Rev. B: Condens. Matter. Mater. Phys*. **50**, 17953−17979 (1994).

8 Katsoulidis, A. P. *et al.* Chemical control of structure and guest uptake by a conformationally mobile porous material. *Nature* **565**, 213-217 (2019).

9 Yamagishi, H. *et al.* Self-assembly of lattices with high structural complexity from a geometrically simple molecule. *Science* **361**, 1242-1246 (2018).

10 Zhan, C. *et al.* A metamorphic inorganic framework that can be switched between eight single-crystalline states. *Nat. Commun.* **8**, 14185 (2017).

11 Song, B.-Q. *et al.* Reversible Switching between Nonporous and Porous Phases of a New SIFSIX Coordination Network Induced by a Flexible Linker Ligand. *J. Am. Chem. Soc.* **142**, 6896-6901 (2020).

12 Piotrowska, R. *et al.* Mechanistic insights of evaporation-induced actuation in supramolecular crystals. *Nat. Mater.* **20**, 403-409 (2021).

13 Zhang, L. *et al.* Hyperexpandable, self-healing macromolecular crystals with integrated

polymer networks. *Nature* **557**, 86-91 (2018).

14 Bradshaw, D. *et al*. Reversible Concerted Ligand Substitution at Alternating Metal Sites in an Extended Solid. *Science* **315**, 977-980 (2007).

15 Zhang, Y.-J., *et al.* Reversible Single-Crystal-to-Single-Crystal Transformation from Achiral Antiferromagnetic Hexanuclears to a Chiral Ferrimagnetic Double Zigzag Chain. *J. Am. Chem. Soc.* **131**, 7942-7943 (2009).

16 Xiao, B. *et al.* Chemically blockable transformation and ultraselective low-pressure gas adsorption in a non-porous metal organic framework. *Nat. Chem.* **1**, 289-294 (2009).

17 Duan, Z. *et al.* Crystal-to-Crystal Transformation from Antiferromagnetic Chains into a Ferromagnetic Diamondoid Framework. *J. Am. Chem. Soc.* **131**, 6934-6935 (2009).

18 Chen, Q. *et al.* A Controllable Gate Effect in Cobalt(II) Organic Frameworks by Reversible Structure Transformations. *Angew. Chem. Int. Ed.* **52**, 11550-11553 (2013).

19 Wu, D.-Q. *et al.* Reversible On–Off Switching of a Single-Molecule Magnet via a Crystal-to-Crystal Chemical Transformation. *J. Am. Chem. Soc.* **139**, 11714-11717 (2017).

20 Zhang, Y. *et al.* Ferroelectricity Induced by Ordering of Twisting Motion in a Molecular Rotor. *J. Am. Chem. Soc.* **134**, 11044-11049 (2012).

21 Phillips, A. E. & Fortes, A. D. Crossover between Tilt Families and Zero Area Thermal Expansion in Hybrid Prussian Blue Analogues. *Angew. Chem. Int. Ed.* **56**, 15950-15953 (2017).
